# Supplementary figures and images for: Compatible interaction of Brachypodium distachyon and endophytic fungus Microdochium bolleyi
Source: PLoS One. 2022 Mar 14;17(3):e0265357. doi: 10.1371/journal.pone.0265357 (PMC8920291; doi:10.1371/journal.pone.0265357)

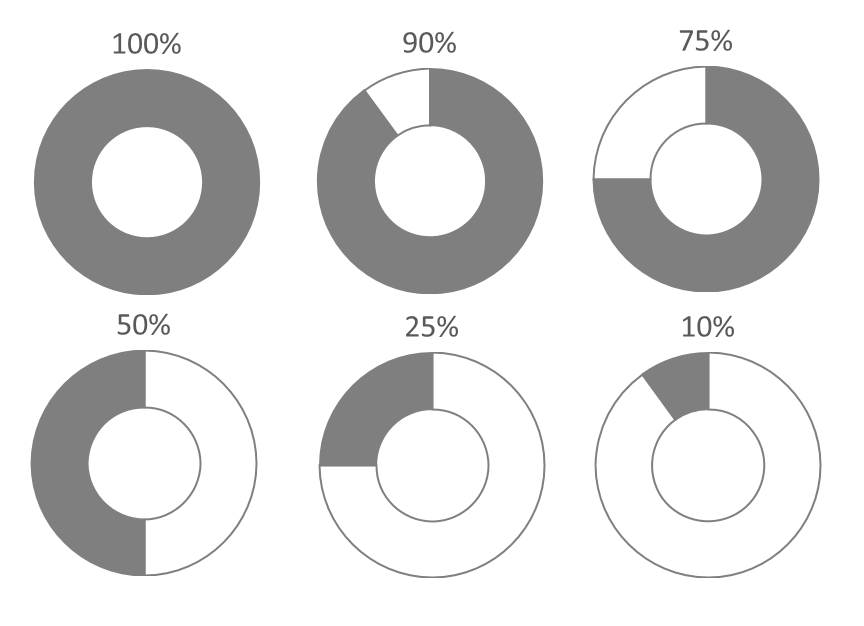

Supplement: S1 Fig — (TIF) [file pone.0265357.s001.tif]

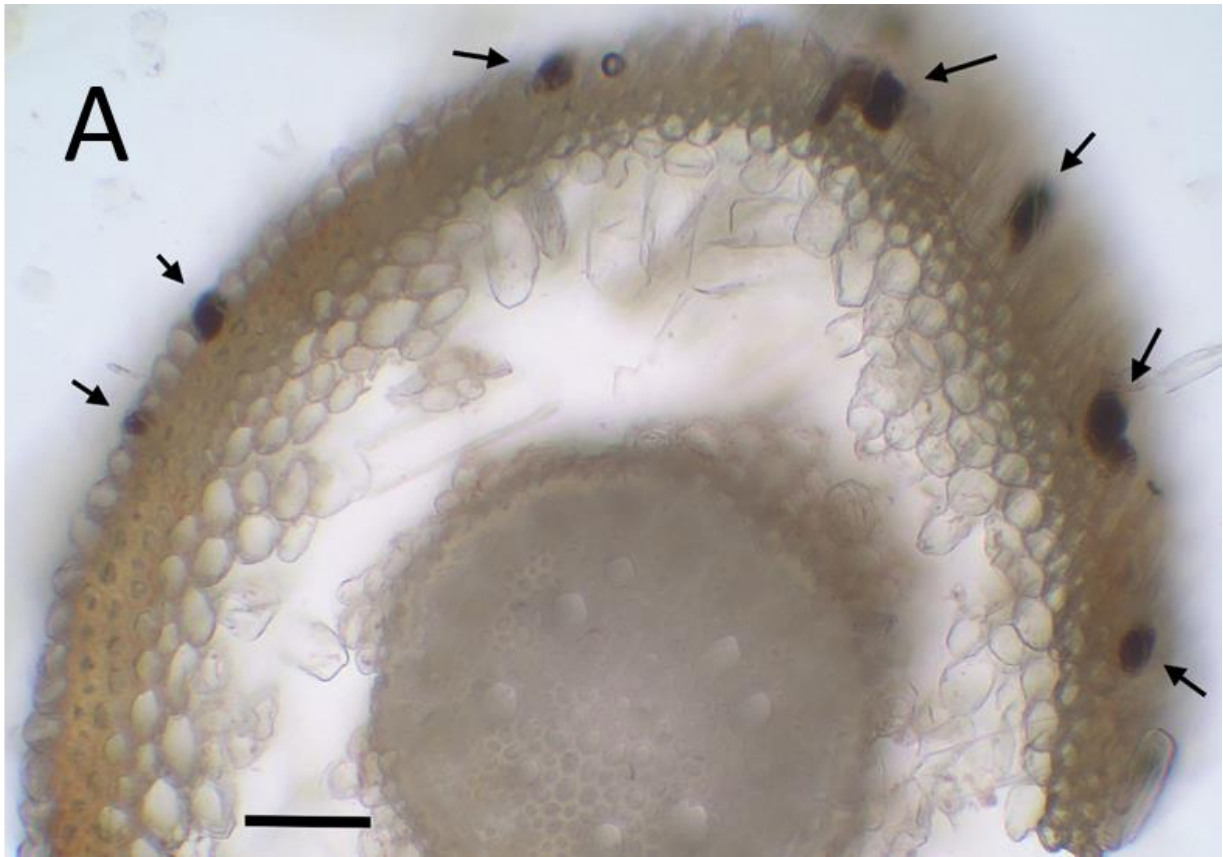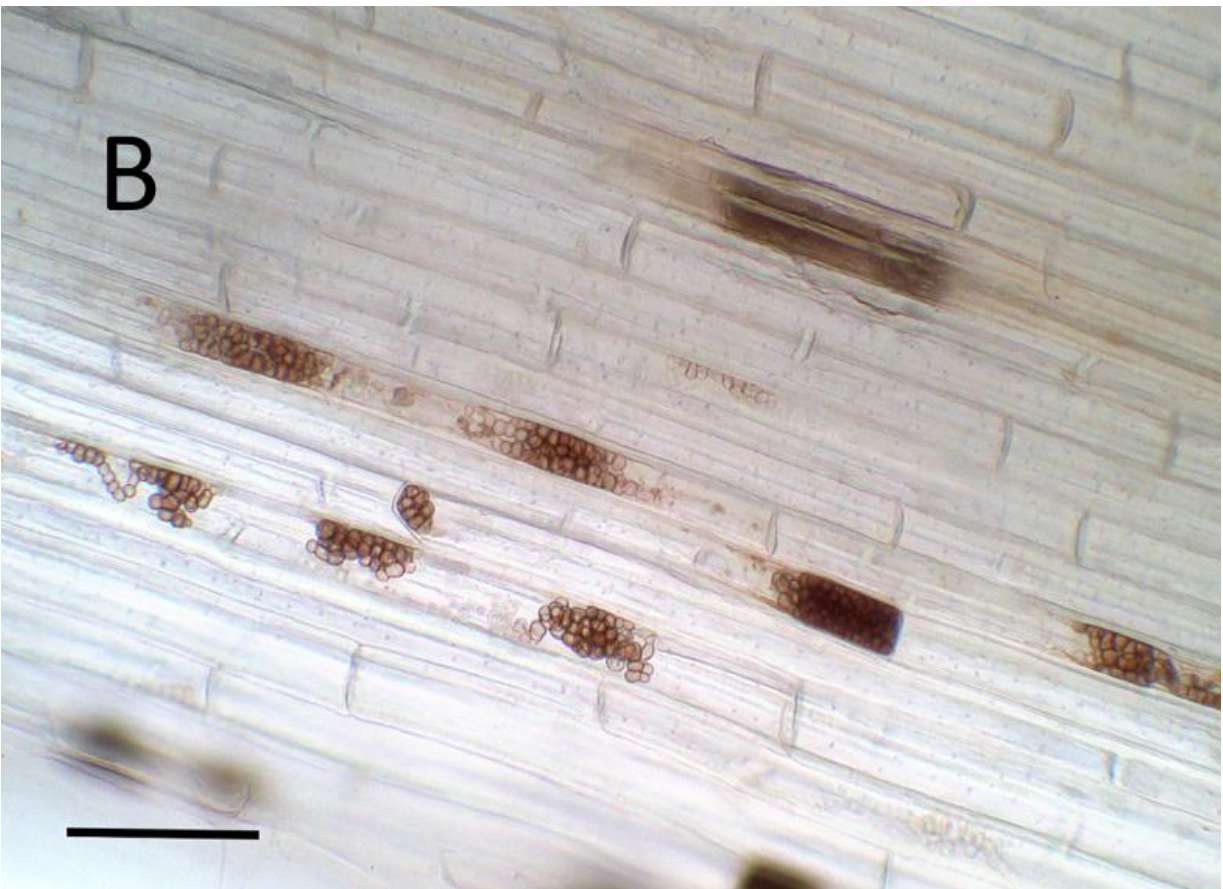

Supplement: S2 Fig — Cross-section by wheat root with cells filled by chlamydospores (arrowed), bar 100µm (A). Wheat rhizodermis with root cells filled by chlamydospores, bar 100µm (B). (PDF) [file pone.0265357.s002.pdf]

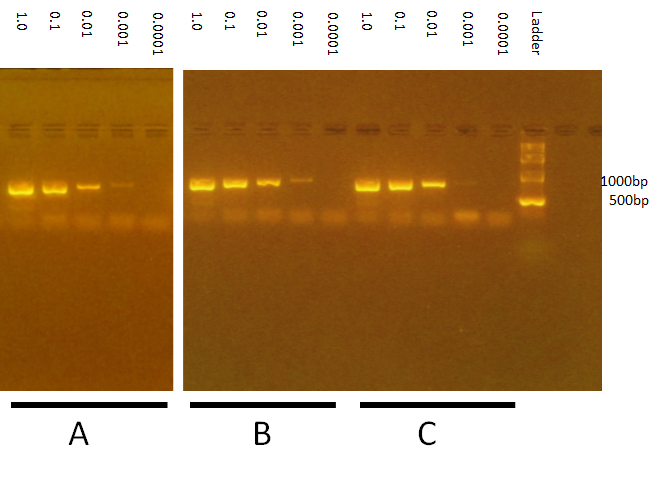

Supplement: S3 Fig — Three different Mb isolates were tested: A) UPOC-FUN-253, B) UPOC-FUN-254, and C) UPOC-FUN-255. (TIF) [file pone.0265357.s003.tif]

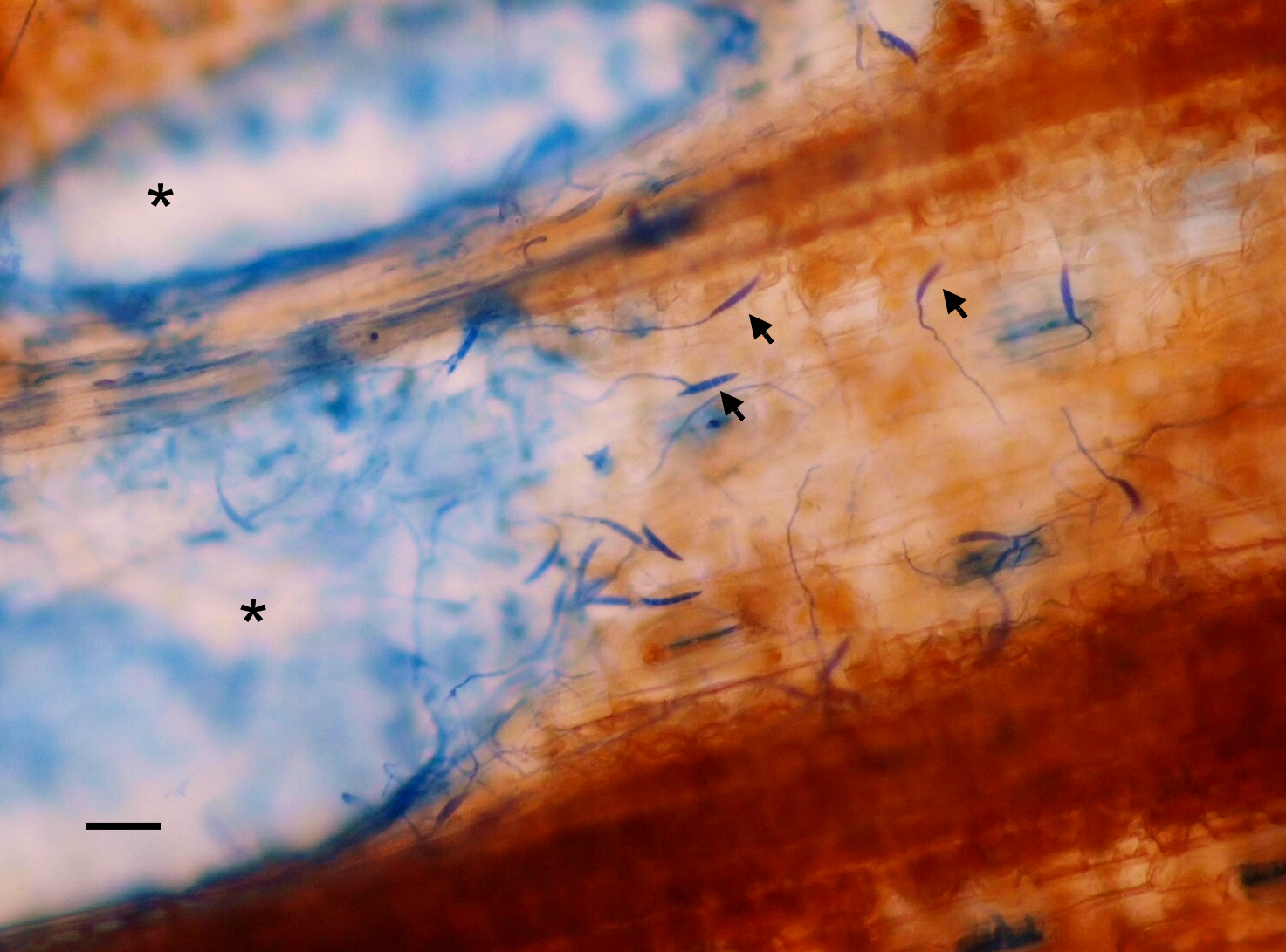

Supplement: S4 Fig — Asterisks indicate light-coloured areas originating from wounding by Pasteur pipette prior to infection. Arrows indicate some germinated macroconidia. (bar = 50 µm). (TIF) [file pone.0265357.s004.tif]

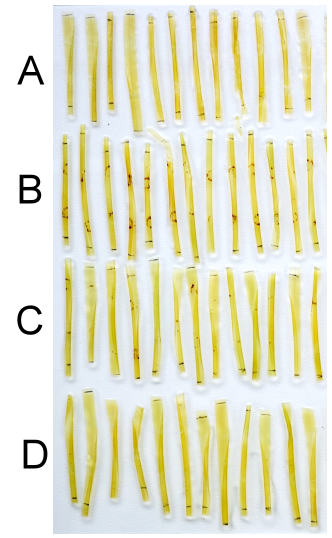

Supplement: S5 Fig — A) Non-inoculated plants with endophyte and without infection by Fc (Mb0F0), B) Non-inoculated plants with endophyte and with infection by Fc (Mb0F1). C) Leaves of plants inoculated with endophyte and infected with Fc (Mb1F1), and D) leaves of plants inoculated with endophyte and without Fc infection (Mb1F0). (TIF) [file pone.0265357.s005.tif]
