# Supplementary material for: Compatible interaction of Brachypodium distachyon and endophytic fungus Microdochium bolleyi
Source: PLoS One. 2022 Mar 14;17(3):e0265357. doi: 10.1371/journal.pone.0265357 (PMC8920291; doi:10.1371/journal.pone.0265357)
Supplement: S1 Table — Names, sequences of forward and reverse primers, publication sources of primer pairs, and gene functions are listed. (DOCX) [file pone.0265357.s006.docx]

**Table S1. Primer pairs used in the study.** Names, sequences of forward and reverse primers, publication sources of primer pairs, and gene functions are listed.

| Gene name | Forward primer | Reverse primer | Publication | Function |
| --- | --- | --- | --- | --- |
| *BdPR1-5* | TACTACACGCACGCCAACAA | GTCGTAGAGGCAGAGCGTG | (1) | Plant defence |
| *BdAOS* | ACCGCCTGGACTTCTACTAC | GAGGTTCTTCTTCTCCACCT | (1) | JA biosynthesis |
| *BdLOX3* | TCAACTTGCCCTTTCCACATG | GCAAACCGGATTAACTCCTGC | (2) | JA biosynthesis |
| *BdChitinase1* | CAGTGGGGCTACTGCTTCAA | GCTGGTAGTTGGACTGCCCT | (3) | Chitinase biosynthesis |
| *BdEIN3* | ATGCTGAATGACAAGTTCCT | AGGTGTAGACACGGTTGTTC | (1) | Ethylene  biosynthesis |
| *BdPAL* | TCTTTGAGGCAAACATTCTT | ATAGCAGCAGCCTCTATTTG | (1) | Phenylalanine ammonia lyase |
| *BdSamDC* | TGCTAATCTGCTCCAATGGC | GACGCAGCTGACCACCTAGA | (3) | reference |
| *TaAOS* | TCCCGAGAGCGCTGTTTAAA | GACGATTGACGGCTGCTATGA | (4) | JA biosynthesis |
| *TaPAL* | TTGATGAAGCCGAAGCAGGACC | ATGGGGGTGCCTTGGAAGTTGC | (5) | Phenylalanine ammonia lyase |
| *TaB2H2* | TCTATCGAAACGCCATTGTTACA | AGAGGCCGTTCGCATAGTCA | (6) | Chitinase biosynthesis |
| *TaPR1.1* | CTGGAGCACGAAGCTGCAG | CGAGTGCTGGAGCTTGCAGT | (7) | Plant defence |
| *TaLOX* | CAGCTGCTCCATGGACTTGA | GCAAGCACGTGATCAACCTG | (8) | JA biosynthesis |
| *TaEIN2* | TGGGTTCATCCAACTGGTC | AAGATGGCATATTGAAATTTG | (9) | Ethylene  biosynthesis |
| *TaGAPDH* | TTAGACTTGCGAAGCCAGCA | AAATGCCCTTGAGGTTTCCC | (10) | reference |
| *MbPOLII* | GCAGGCTTGTGGTCTGGTCA | GTTCGGCTCCTCGCTGTTATCC | Current study | RNA polymerase II second LSU |

1. **Kouzai Y, Kimura M, Yamanaka Y, Watanabe M, Matsui H, Yamamoto M, Ichinose Y, Toyoda K, Onda Y, Mochida K, Noutoshi Y.** Expression profiling of marker genes responsive to the defence-associated phytohormones salicylic acid, jasmonic acid and ethylene in *Brachypodium distachyon*. BMC Plant Biol. 2016; 16(59):1–11.

2. **Sandoya GV, Buanafina de Oliveira MM.** Differential responses of *Brachypodium distachyon* genotypes to insect and fungal pathogens. Physiol Mol Plant Pathol. 2014; 85:53–64.

3. **Hong SY, Seo PJ, Yang MS, Xiang F, Park CM.** Exploring valid reference genes for gene expression studies in Brachypodium distachyon by real-time PCR. BMC Plant Biol. 2008;7(8):112.

4. **Liu X, Meng J, Starkey S, Smith C.** Wheat gene expression is differentially affected by a virulent Russian wheat aphid biotype. J Chem Ecol. 2011;37:472–482.

5. **Ding L, Xu H, Yi H, Yang L, Kong Z, Zhang L, et al.** Resistance to hemi-biotrophic *F. graminearum* infection is associated with coordinated and ordered expression of diverse defense signaling pathways. PLoS One*.* 2011; 4:e19008.

6. **Kong L, Anderson JM, Ohm HW.** Induction of wheat defense and stress-related genes in response to *Fusarium graminearum*. Genome*.* 2005;48(1): 29–40.

7. **Molina A, Görlach J, Volrath S, Ryals J.** Wheat genes encoding two types of PR-1 proteins are pathogen inducible, but do not respond to activators of systemic acquired resistance. *Mol Plant Microbe Interact.* 1999;12(1):53–58.

8. **Zhang, J, Yan H, Xia M.** Wheat root transcriptional responses against *Gaeumannomyces graminis* var. *tritici*. Phytopathol Res. 2020;2(23):1–14.

9. **Travella S, Klimm TE, Keller B.** RNA interference-based gene silencing as an efficient tool for functional genomics in hexaploid bread wheat. Plant Physiol*.* 2006;142: 6–20.

10. **Sun H, Guo Z, Gao L, Zhao G, Zhang W, Zhou R, et al.** DNA methylation pattern of Photoperiod-B1 is associated with photoperiod insensitivity in wheat (*Triticum aestivum*). New Phytol*.* 2014;204(3):682–692.
