# Supplementary material for: Compatible interaction of Brachypodium distachyon and endophytic fungus Microdochium bolleyi
Source: PLoS One. 2022 Mar 14;17(3):e0265357. doi: 10.1371/journal.pone.0265357 (PMC8920291; doi:10.1371/journal.pone.0265357)
Supplement: S2 Table — The microscopic method was based on the presence of chlamydospores in roots after staining with aniline blue. PCR methods were performed using MbITSRTF/R primers. (DOCX) [file pone.0265357.s007.docx]

**Table S2. Microscopic and molecular detection of Mb in Bd and wheat roots.** The microscopic method was based on the presence of chlamydospores in roots after staining with aniline blue. PCR methods were performed using MbPOLII primers.

| Species | Inoculated by endophyte | N | Number of plants with positive detection of Mb | |
| --- | --- | --- | --- | --- |
|  |  |  | Microscopic | PCR |
| Bd | Mb1 (yes) | 30 | 30 | 30 |
|  | Mb0 (no) | 30 | 0 | 0 |
| Wheat | Mb1 (yes) | 30 | 30 | 30 |
|  | Mb0 (no) | 30 | 0 | 0 |
