# Supplementary material for: Compatible interaction of Brachypodium distachyon and endophytic fungus Microdochium bolleyi
Source: PLoS One. 2022 Mar 14;17(3):e0265357. doi: 10.1371/journal.pone.0265357 (PMC8920291; doi:10.1371/journal.pone.0265357)
Supplement: S3 Table — (DOCX) [file pone.0265357.s008.docx]

**Table S3. ANOVA statistical evaluation for damage to leaves of *Brachypodium distachyon* by pathogen *Fusarium culmorum* when the main experimental factor is previous inoculation with the endophytic fungus *Microdochium bolleyi*.**

| Source of variation | Sum of squares | Degrees of freedom | Mean square | *F*-statistic | *p*-value |
| --- | --- | --- | --- | --- | --- |
| Endophyte | 21093.75 | 1 | 21093.75 | 27.28838 | 0.000003 |
| Replication | 1669.17 | 4 | 417.29 | 0.53984 | 0.707088 |
| Error | 41741.67 | 54 | 772.99 |  |  |
