# Supplementary material for: Compatible interaction of Brachypodium distachyon and endophytic fungus Microdochium bolleyi
Source: PLoS One. 2022 Mar 14;17(3):e0265357. doi: 10.1371/journal.pone.0265357 (PMC8920291; doi:10.1371/journal.pone.0265357)
Supplement: S4 Table — (DOCX) [file pone.0265357.s009.docx]

**Table S4. ANOVA statistical evaluation for damage to leaves of wheat by pathogen *Fusarium culmorum* when the main experimental factor is previous inoculation with the endophytic fungus *Microdochium bolleyi*.**

| Source of variation | Sum of squares | Degrees of freedom | Mean square | *F*-statistic | *p*-value |
| --- | --- | --- | --- | --- | --- |
| Endophyte | 13801.67 | 1 | 13801.67 | 15.31077 | 0.000257 |
| Replication | 2414.17 | 4 | 603.54 | 0.66953 | 0.615949 |
| Error | 48677.50 | 54 | 901.44 |  |  |
